# Ozone and nitrogen dioxide regulate similar gene expression responses in Arabidopsis but natural variation in the extent of cell death is likely controlled by different genetic loci

**DOI:** 10.3389/fpls.2022.994779

**Published:** 2022-10-19

**Authors:** Johanna Leppälä, Frank Gaupels, Enjun Xu, Luis O. Morales, Jörg Durner, Mikael Brosché

**Affiliations:** ^1^ Organismal and Evolutionary Biology Research Programme, Viikki Plant Science Centre, Faculty of Biological and Environmental Sciences, University of Helsinki, Helsinki, Finland; ^2^ Institute of Biochemical Plant Pathology, Helmholtz Zentrum München, German Research Center for Environmental Health, Neuherberg, Germany

**Keywords:** genome wide association study, cell death, ozone, nitrogen dioxide, gene expression, stress responses

## Abstract

High doses of ozone (O_3_) and nitrogen dioxide (NO_2_) cause damage and cell death in plants. These two gases are among the most harmful air pollutants for ecosystems and therefore it is important to understand how plant resistance or sensitivity to these gases work at the molecular level and its genetic control. We compared transcriptome data from O_3_ and NO_2_ fumigations to other cell death related treatments, as well as individual marker gene transcript level in different *Arabidopsis thaliana* accessions. Our analysis revealed that O_3_ and NO_2_ trigger very similar gene expression responses that include genes involved in pathogen resistance, cell death and ethylene signaling. However, we also identified exceptions, for example *RBOHF* encoding a reactive oxygen species producing RESPIRATORY BURST OXIDASE PROTEIN F. This gene had increased transcript levels by O_3_ but decreased transcript levels by NO_2_, showing that plants can identify each of the gases separately and activate distinct signaling pathways. To understand the genetics, we conducted a genome wide association study (GWAS) on O_3_ and NO_2_ tolerance of natural Arabidopsis accessions. Sensitivity to both gases seem to be controlled by several independent small effect loci and we did not find an overlap in the significantly associated regions. Further characterization of the GWAS candidate loci identified new regulators of O_3_ and NO_2_ induced cell death including ABH1, a protein that functions in abscisic acid signaling, mRNA splicing and miRNA processing. The GWAS results will facilitate further characterization of the control of programmed cell death and differences between oxidative and nitrosative stress in plants.

## 1 Introduction

Ozone (O_3_) and nitrogen dioxide (NO_2_) are common air pollutants that occur in the troposphere, the lowest part of the Earth’s atmosphere. In high concentrations, they cause damage to plants and animals (Middleton, 1961; Brunekreef and Holgate, 2002; Ainsworth, 2017). O_3_ and nitrogen oxides are amongst the three most harmful air pollutants in terms of damage to ecosystems (The European Environment Agency, 2020). Low to intermediate ppb (parts per billion) levels of O_3_ are harmful to plants (McGrath et al., 2015). However, NO_2_ is less toxic than O_3_, causing reduced plant growth and lesion formation at concentrations in the high ppb to low ppm (parts per million) range (Taylor and Eaton, 1966; Kasten et al., 2016). Several studies indicate large-scale yield loss due to O_3_ pollution in agriculturally important species, including maize, soybean, rice and wheat (McGrath et al., 2015; Feng et al., 2022b).

The mechanisms leading to damage and cell death after exposure to high levels of O_3_ are better understood than those of NO_2_. Both gases enter the plants through the stomatal pores. In the apoplastic space, O_3_ forms reactive oxygen species (ROS), including superoxide and hydrogen peroxide (Vainonen and Kangasjarvi, 2015). Whether plants tolerate the amount of ROS produced by O_3_ or initiate cell death depends on antioxidant capacity and the balance of stress hormone signaling. Increased production of ethylene promotes O_3_ induced cell death in several plant species (Tuomainen et al., 1997; Overmyer et al., 2000; Vahala et al., 2003), whereas jasmonic acid (JA) protects from O_3_ damage (Rao et al., 2000; Xu et al., 2015a). Salicylic acid (SA) is involved in both promotion of O_3_ induced cell death as well as activation of defence response to O_3_ (Rao and Davis, 1999; Xu et al., 2015b). The role of hormones in O_3_ responses has been studied with *A. thaliana* mutants such as the JA receptor mutant *coi1*, and the ethylene overproducing mutant *eto1*, that are both O_3_ sensitive (Rao et al., 2002; Xu et al., 2015a). After entering the apoplast, NO_2_ rapidly reacts with water to produce nitrate, nitrite, nitric oxide (NO), and protons. Especially nitrite and NO are important drivers of NO_2_ induced cell death in *A. thaliana* (Kasten et al., 2016). Mutant analyses suggested that signaling by SA enhanced NO_2_ tolerance whereas JA does not regulate NO_2_ induced cell death (Kasten et al., 2016). Small molecule antioxidants such as ascorbic acid (vitamin C) are important scavengers of ROS and RNS in plant tissues. Accordingly, the ascorbic acid deficient mutant *vtc1* is sensitive to both O_3_ as well as NO_2_ (Conklin et al., 1996; Kasten et al., 2016).

Plants produce ROS and reactive nitrogen species (RNS) as second messengers to regulate growth and development, stress responses and long-distance signaling (Waszczak et al., 2018; Hancock and Neill, 2019; Castro et al., 2021). In plant-pathogen signaling, both ROS and NO acts as signal molecules, and are proposed to participate in an amplification loop to enhance signaling (Wang et al., 2018). Further, the balance between ROS and NO signaling regulates programmed cell death (PCD) (Delledonne et al., 2001; Wendehenne et al., 2014) Therefore, at least some effects of O_3_ and NO_2_ (or their reactive derivatives) relates to activation of cellular signaling processes. This assumption is supported by increased transcript levels of pathogen responsive and PCD related genes by both O_3_ and NO_2_ (Xu et al., 2015a; Mayer et al., 2018). Consequently, exposure to these gasses leads to the establishment of basal pathogen resistance in *A. thaliana* against the bacterial pathogen *Pseudomonas syringae* (Sharma et al., 1996; Mayer et al., 2018). Infection with avirulent pathogens elicit the hypersensitive defence response (HR) culminating in localized PCD, thought to restrict pathogen growth (Delledonne et al., 2001; Gaupels et al., 2011; Wang et al., 2013). O_3_- and NO_2_-triggered cell death is remarkably similar to HR-PCD since it is also dependent on simultaneous signaling by NO and ROS and is accompanied by the accumulation of fluorescent compounds in dying leaf tissues (Overmyer et al., 2005; Ahlfors et al., 2009; Kasten et al., 2016). Hence, O_3_ and NO_2_ cause leaf damage at least partially by activation of PCD (Gandin et al., 2021; Hussain et al., 2022).

Although the damaging effects of O_3_ and NO_2_ have been assessed in several studies, much less is known about the genetic factors controlling tolerance of plants to these pollutants. Regulation of O_3_ and NO_2_ responses in *A. thaliana* has mostly been studied in the genetic background Col-0 (Overmyer et al., 2008; Frank et al., 2019). Natural accessions of *A. thaliana* show large variation in their O_3_ sensitivity (Brosche et al., 2010). For instance, Cvi-0 is very sensitive but Col-0 rather tolerant to 300-350 ppb O_3_ for 6 h (Brosche et al., 2010). By studying naturally occurring variation for stress tolerance in multiple accessions, instead of a mutant approach in a single genetic background, it is possible to gain broader understanding of the underlying genetics. Natural variation can be explored with a genome wide association study (GWAS). This approach takes advantage of naturally occurring genetic recombination events to associate phenotypes of accessions with single nucleotide polymorphisms (SNPs) in close genomic vicinity of causative genes. The 1001 Genomes project has provided genome sequences of more than a thousand natural *A. thaliana* accessions and this data is available for use as genetic markers for GWAS (Alonso-Blanco et al., 2016).

To further understand how O_3_ and NO_2_ regulates defence signaling and cell death, we used two complementary approaches; transcriptome analysis and GWAS. The transcriptional responses to O_3_ and NO_2_ treatments were previously analysed individually (Brosche et al., 2014; Xu et al., 2015a; Mayer et al., 2018), and here we systematically identify the regulatory context of O_3_ and NO_2_. In a comparison of different transcriptome datasets, we found very high overlaps of differentially expressed genes regulated by O_3_ and NO_2_. With real time quantitative PCR (qPCR) we confirmed that both gasses activated similar signaling in different *A. thaliana* accessions. However, we also identified a marker gene with differential O_3_ versus NO_2_ response, demonstrating that *A. thaliana* can activate precise signal activation to each gas. We continued with GWAS for O_3_ and NO_2_ leaf damage with up to 372 A*. thaliana* natural accessions. We identified 12 genomic loci associated with O_3_ and NO_2_ induced leaf damage. Experiments with T-DNA knock-out mutants suggest functions of several GWAS-derived candidate genes in O_3_ and NO_2_ sensitivity.

## 2 Materials and methods

### 2.1 Plant material

Altogether 372 A*. thaliana* accessions (**Supplementary Table S1**) were selected so that their genotypes were available from 250k SNP chip (Atwell et al., 2010). The accessions were a subset of the 1135 accessions sequenced by the 1001 Genomes Consortium (http://1001genomes.org/), and the population structure was described earlier (Alonso-Blanco et al., 2016). The seeds for natural accessions were obtained from the Nottingham Arabidopsis Stock Centre (**Supplementary Table S1**). Mutant seeds were obtained from the Nottingham Arabidopsis Stock Centre or were donated by Dr. Patricia Conklin (*vtc1-*1), Dr. Heribert Hirt (*mpk6*), Dr. John Turner (*coi1-16*) or were EMS mutants from Helsinki (*rcd1-1*, *slac1-1* (Overmyer et al., 2000; Vahisalu et al., 2008)).

### 2.2 Growth conditions

Experiments were performed in Helsinki and Munich. Plants in Helsinki were grown in 1:1 peat-vermiculite under 280 µmol m^2^ s^-1^ white light irradiance, 12 h : 12 h light-dark –cycle, 23°C/19°C (day/night) temperature and 70%/90% relative humidity. In Munich plants were grown in 5:1 Floradur propagation substrate - quartz sand, in walk-in size chambers, under 250 μmol m^2^ s^-1^; PAR, 12 h : 12 h light-dark –cycle, 23°C/18°C (day/night) temperature and 70%/90% relative humidity. Plants grew faster in Munich than in Helsinki, possibly due to small differences in light and soil quality. However, plants were exposed to O_3_ in Munich and Helsinki at a similar developmental stage (estimated by counting leaf numbers of the control plants Col-0 and Cvi-0). Therefore, plants were treated with O_3_ approximately 4 days younger in Munich than in Helsinki.

#### 2.2.1 O_3_ fumigations in Helsinki

372 accessions at the age of 23 days were exposed to 400 ppb of O_3_ for 6 has described previously (Brosche et al., 2010). All treatments were started in the morning after approximately 2 h of light exposure. There were 8 plants of each genotype in one replicate. 23 accessions with controls could be treated simultaneously. Each genotype was present in two to three replicates. The O_3_ phenotype was scored as number of injured leaves from all leaves relative to damage in Col-0.

#### 2.2.2 O_3_ fumigations in Munich

All 127 accessions were treated simultaneously in a single fumigation chamber. Five 19 days-old plants of every accession were fumigated for 6 h with 350 ppb of O_3_. The experiment was replicated twice.

#### 2.2.3 NO_2_ fumigations in Munich

Three 24-28 days-old plants of 216 accessions were exposed to NO_2_ for 1 h in an air-tight fumigation chamber as reported earlier (Frank et al., 2019). Plants were short-term fumigated for 1 h with 10, 20 and 30 ppm of NO_2_. This range of concentrations was chosen as sensitive plants were damaged already with 10 ppm whereas tolerant plants displayed only weak symptoms even after 30 ppm of NO_2_. The NO_2_ phenotype was scored as percent leaf area damaged (0% = score 1, <25% = score 2, 25-50% = 3, 50-75% = 4, >75% = 5). A cumulative score (scale from 3 to 15) from all three fumigations was used in further analyses. The experiment was repeated three times.

### 2.3 Analysis of public gene expression data

Hierarchical clustering was done with publicly available data (**Supplementary Table S2**). The raw data were processed with robust multiarray average normalization using Bioconductor limma and affy packages in R. Gene expression was summarized by calculating log_2_ ratio of the treatment/mutant and control/wild type expression. Bayesian hierarchical clustering method was used (as described in (Wrzaczek et al., 2010)) with 1000 bootstrap resampling.

To compare similarities in O_3_ and NO_2_ transcriptomes we used data from exposures of *A. thaliana* Col-0 to 350 ppb O_3_ for 2 h (RNA-seq analysis) (Xu et al., 2015a; Xu et al., 2015b) and 10 ppm NO_2_ for 1 h (microarray analysis) (Mayer et al., 2018), or a NO experiment with the donor S-nitrosocysteine (1 mM, 6 h timepoint) (Hussain et al., 2016). Analysis of RNA-seq data is described in the **Supplementary Methods**. GO-term enrichment was performed in R (R Core Team, 2018), version 3.5.0, using the package clusterProfiler (Yu et al., 2012). Venn diagrams were constructed using jvenn software (Bardou et al., 2014).

### 2.4 Real time reverse transcriptase quantitative PCR (qPCR)

Nine accessions were grown for qPCR analysis in Munich facilities in similar environmental and soil conditions as for GWAS. Plants were treated with 350 ppb O_3_ or 10 ppm NO_2_ at the age of three weeks. Three plants per genotype were collected and pooled in liquid nitrogen 2 h after the start of fumigations. RNA isolation, cDNA synthesis and qPCR were performed as described (Xu et al., 2015a). Normalization of the data was performed in qBase 2.0 [Biogazelle, (Hellemans et al., 2007)], with three reference genes *SAND*, *TIP41* and *YLS8* (Czechowski et al., 2005) The whole experiment was replicated three times. Primer sequences and amplification efficiencies can be found in **Supplementary Table S3**.

### 2.5 Genome-wide association studies

The GWA analyses were performed for the maximum numbers of phenotyped accessions for each trait, as well as separately for the set of accessions that was shared between all three experiments (119 accessions). The phenotype data from NO_2_ treatment was nearly normally distributed but the phenotype data from both O_3_ experiments were skewed as there were more tolerant accessions compared to sensitive. No transformations were used as these did not provide normality for the phenotypic data. Before the full genome data was available, the O_3_ datasets were analysed with 250K SNP array data (Kim et al., 2007; Atwell et al., 2010) with EMMAX (Kang et al., 2010), from where some candidate genes were identified based on their biological function. The main GWA analyses of the damage screens were conducted in GWA-portal (http://gwas.gmi.oeaw.ac.at/, predecessor GWAPP by (Seren et al., 2012)), where imputed full genome data (Cao et al., 2011; Gan et al., 2011; Long et al., 2013), could be used for association analysis. The numbers of investigated SNPs were 4.1 million for 119 common accessions (O_3_ and NO_2_), 5.5 million for 372 accessions (O_3_ Helsinki), 4.3 million for 127 accessions (O_3_ Munich) and 4.9 million for 216 accessions (NO_2_). The average SNP density in our GWA analyses was around one SNP per 25 base pairs, which should provide a very good coverage of the genome, even though linkage disequilibrium decays rapidly (on average within 10 kb (Kim et al., 2007)). We did not perform filtering based on minor allele frequency, but included all the SNPs in the analysis, as we wanted to include rare variants that play a role in O_3_ sensitivity (Jakobson et al., 2016). Both non-parametric Kruskal-Wallis and accelerated mixed model (AMM) approaches were used. As variants truly associated with the traits of interest may occur in certain populations and therefore be correlated with population structure, we present data from Kruskal-Wallis tests, which does not correct for the population structure. AMM uses a linear mixed model approach that was developed by Kang et al. which corrects for population structure and genetic relatedness in association mapping (Kang et al., 2008; Kang et al., 2010).

### 2.6 Ion leakage of T-DNA mutant lines

To verify genomic regions identified by GWA, T-DNA lines (in Col-0 accession) of candidate genes were tested for O_3_ and NO_2_ sensitivity. As candidate genes we included all the genes that had SNPs in high linkage disequilibrium (r^2^ > 0.8) with the Bonferroni corrected significant SNPs, as well as two genes that were selected based on their biological functions from the analysis with 250K SNP array data (**Table 1**). The mutant lines are described in more detail in **Supplementary Table S4**. The T-DNA mutant lines were selected to have inserts in exons and were confirmed by PCR to be homozygous for the insert (PCR primers used for genotyping in **Supplementary Table S4**). Plants for ion leakage experiments were grown as described earlier and fumigated with O_3_ (350 ppb, 6 h) in Helsinki and NO_2_ (10 ppm for 6 h or 30 ppm for 1 h) in Munich. Ion leakage was performed as earlier (Brosche et al., 2014; Kasten et al., 2016). The experiments were repeated at least three times. Statistical analysis of the ion leakage measurements was done with linear mixed models in R 3.4.3 (R Core Team, 2018), with *lme4* package. As several mutant lines were compared to the same control (Col-0), we used Dunnett’s test, with *multcomp* package, to evaluate which comparisons were significant.

**Table 1:** Identified GWAS candidate genes, -log10 P-value of the most significant SNP in the regions, minor allele frequency and count of the most significant SNPs, significant differences in ion leakage measurements of the candidate gene mutants relative to Col-0 control after NO2 and O3 fumigations.

Treatment	Number of accessions	Experiment location	GWA analysis	Locus	Gene name or description	-log10 P-value	MAF	MAC	Ion leakage 10 ppm NO_2_	Ion leakage 350 ppb O_3_	T-DNA line
O_3_	119	Helsinki	AMM	AT3G61410	U-box kinase	8,36	0,1	12	–	–	–^a^
O_3_	119	Munich	AMM	AT2G43790	MPK6	8,43	0,17	20	ns	ns	SALK_073907
				AT2G43795					ns	ns	SALK_112469
O_3_	372	Helsinki	AMM	AT1G44890	inner membrane OXA1-like protein	8,82	0,01	3	ns	ns	GK-111B06
				AT1G44900	MCM2				–	–	–^b^
				AT1G44910	PRP40A				–	–	–^b^
O_3_	372	Helsinki	AMM	AT2G38620	CDKB1;2	9,12	0,02	9	ns	ns	SALK_133560C
				AT2G38630	Transducin/WD40 repeat-like superfamily protein				ns	ns	SAIL_792_C08
				AT2G38640	LURP-one-like protein				-30%, **	ns	SAIL_198_B02
				AT2G38650	GAUT7				ns	ns	SALK_015189
O_3_	372	Helsinki	AMM	AT5G23220	NIC3	9,04	0,01	4	–	–	–^c^
O_3_	372	Helsinki	AMM, 250K	AT5G53130	CNGC1	5,13	0,24	84	ns	+35%, ***	SAIL_443_B11
O_3_	127	Munich	AMM	AT3G53400		8,06	0,06	8	+23%, **	ns	SALK_206949C
O_3_	127	Munich	KW, 250K	AT2G13540	ABH1	3,68	0,21	26	ns	+142%, ***	SALK_024285
NO_2_	216	Munich	KW	AT1G19480	DNA glycosylase superfamily protein	8,44	0,13	27	ns	+30%, ***	SALK_022386
				AT1G19485	Transducin/WD40 repeat-like superfamily protein				+32%, *	ns	SALK_076362
				AT1G19490	Basic-leucine zipper (bZIP) transcription factor				ns	ns	SALK_053908C
NO_2_	216	Munich	KW	AT2G03740	LEA11	8,25	0,34	74	–	–	–^c^
NO_2_	216	Munich	KW	AT4G32105	Beta-1,3-N-Acetylglucosaminyltransferase family protein	8,01	0,39	84	–	–	–^b^
NO_2_	216	Munich	KW	AT5G08005	flavonoid protein	8,2	0,28	61	–	–	–^c^
				AT5G08010					–	–	–^a^

MAF, minor allele frequency; MAC, minor allele count; Bonferroni corrected P-values: ***p < 0.001, **p < 0.01, *p < 0.05. ns, not significant; NA, not available.

^a^T-DNA line ordered but only wild type plants found after PCR testing; ^b^no homozygous T-DNA line where insert in exon; ^c^no T-DNA line where insert in exon available.

## 3 Results

### 3.1 O_3_ and NO_2_ trigger the expression of genes related to cell death and pathogen resistance

**Figure 1:**
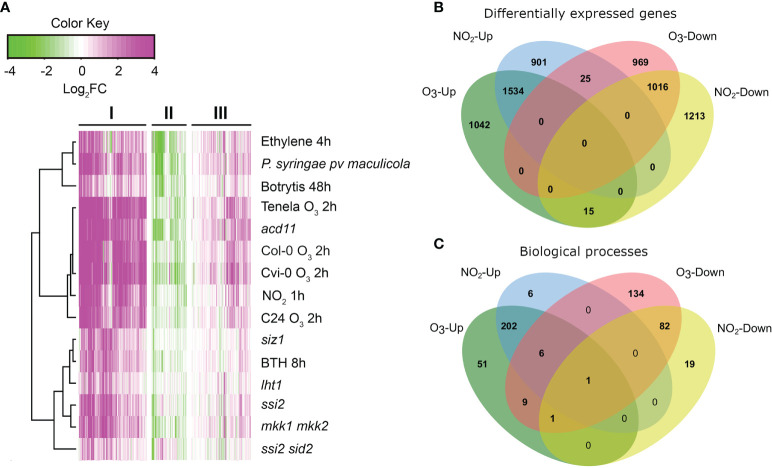
Comparison of O_3_ and NO_2_ transcriptional profiles. **(A)** Bayesian hierarchical clustering of genes from the gene ontology category cell death. Datasets were selected from NO_2_ and O_3_ treatments, pathogen and hormone treatments, and lesion mimic mutants that develop PCD lesions (see **Supplementary Table 2** for more details). Values are log_2_ -transformed fold changes in gene expression. Magenta and green indicate increased and decreased (respectively) gene expression compared to untreated or wild type control plants. **(B)** Comparison of differentially expressed genes after O_3_ 2 h and NO_2_ 1 h (see also **Supplementary Table S5**). **(C)** Overlap in significantly enriched GO Biological processes between O_3_ and NO_2_.

ROS and RNS act as signals in plant stress responses, pathogen resistance, and PCD (Delledonne et al., 2001; Wang et al., 2013; Xu et al., 2015a; Mayer et al., 2018). To place O_3_- and NO_2_-induced transcriptome changes into the context of PCD, we performed Bayesian hierarchical clustering with all genes from the gene ontology (GO) category cell death (**Figure 1A**). Transcriptome datasets included both air pollutants, cell death in lesion mimic mutants, pathogen infection and hormone treatments (for a full list of experiments see **Supplementary Table S2**). The three resulting clusters (**Figure 1A**) contained genes with strongly increased (cluster I), decreased (II), or weakly increased (III) transcript levels. In cluster I, there were large similarities between exposure to 350 ppb O_3_ (2 h), 10 ppm NO_2_ (1 h), pathogen infections (*Botrytis cinerea*, *Pseudomonas syringae pv. maculicola*) and lesion mimic mutants that undergo spontaneous cell death (*acd11*, *mkk1 mkk2*, *siz1*, *ssi2*). This highlights that the O_3_ and NO_2_ treatments trigger similar signaling pathways as those activated during pathogen infection and cell death.

To explore the overlap in transcriptional regulation between O_3_ and NO_2_, we used transcriptome data from exposures of *A. thaliana* Col-0 to 350 ppb O_3_ for 2 h (Xu et al., 2015b) and 10 ppm NO_2_ for 1 h (Mayer et al., 2018) (**Supplementary Table S5**). In this comparison 1534 genes were >2-fold up- and 1016 genes down-regulated by both gases (**Figure 1B**), i.e >55% of the genes regulated by NO_2_ were also responsive to O_3_ and vice versa. GO term enrichment analysis revealed that O_3_ and NO_2_ activated similar biological processes (**Figure 1C**) including “regulation of immune response”, “regulation of plant-type hypersensitive response”, “respiratory burst”, and “response to ethylene”. In sum, O_3_ and NO_2_ transcriptionally regulate largely overlapping sets of genes involved in pathogen resistance, cell death, and ethylene signaling.

As NO is a main component in RNS signaling (Delledonne et al., 2001; Kasten et al., 2016), we also made transcriptome comparisons between O_3_ and treatment with the NO-donor S-nitrosocysteine (Hussain et al., 2016). Similar to the O_3_-NO_2_ comparison, O_3_ and S-nitrosocysteine regulated genes showed a large overlap with more than 6000 genes regulated by both treatments (**Supplementary Figure S1A**). GO enrichment showed that many aspects of defense signaling (including “phosphorelay signal transduction system”, “response to ROS” and “regulation of response to biotic stimulus”) were found among the genes with increased expression by both treatments (**Supplementary Table S6**).

### 3.2 O_3_- and NO_2_-induced defence transcriptional responses is similar between *A. thaliana* natural accessions, with notable exceptions

One drawback in the re-analysis of transcriptome data from different laboratories is that the experiments are heterogeneous in terms of plant growth conditions and treatments. Moreover, the transcriptome data shown in **Figure 1** mainly originate from experiments with *A. thaliana* Col-0, and thus, might not reflect the natural variation within *A. thaliana* accessions. To address these issues, we grew and treated a set of accessions under controlled conditions followed by transcript level analysis using real time quantitative PCR (qPCR). The selected accessions covered different O_3_ sensitivities (e.g. Ts-1 is O_3_ tolerant and Cvi-0 O_3_ sensitive), natural habitats, and genetic distances (Cvi-0 is more distantly related to other accessions [Alonso-Blanco et al., 2016)]. The plants were treated with 350 ppb O_3_ and 10 ppm NO_2_ as these treatments induced largely overlapping sets of genes in Col-0 (**Figure 1**).

**Figure 2:**
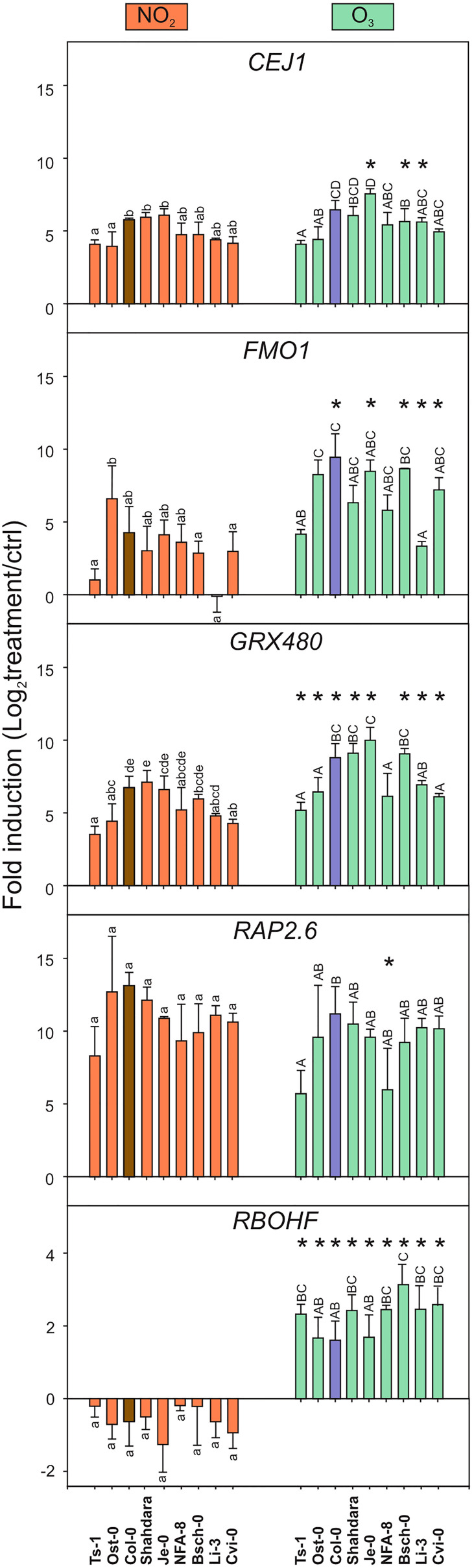
Transcript levels after NO_2_ and O_3_ treatments in different accessions. Transcript levels of five marker genes was measured with qPCR in natural accessions of *A. thaliana* 2* h* after treatments with NO_2_ (left column) and O_3_ (right column). Log_2_ –transformed fold change of marker genes in natural accessions are shown from three biological replicates. Statistical analysis was performed with a linear mixed model (*P* < 0.05). Letters from a to e represent comparison of NO_2_ treatment effect, and letters from A to D O_3_ treatment effect, on different genotypes. Asterisks (*) represent statistical differences between NO_2_ and O_3_ treatments on each genotype. Error bars display standard deviation. The accessions were selected to include genotypes with differential NO_2_ and O_3_ damage phenotypes (see also **Figure 3A**).

For qPCR analysis (**Figure 2**), we selected marker genes from the GO category cell death that also has been used as marker genes for the stress hormones SA, JA and ethylene. The ethylene/JA marker genes *CEJ1* (*COOPERATIVELY REGULATED BY ETHYLENE AND JASMONATE 1*) and *RAP2.6* (*RELATED TO AP2.6* (Krishnaswamy et al., 2011)) had increased transcript levels and were regulated similarly by both gases in all accessions. The SA marker gene *GRX480* (*GLUTAREDOXIN 480*; (Caarls et al., 2015) and systemic signaling *FMO1* (*FLAVIN-DEPENDENT MONOOXYGENASE 1* (Hartmann et al., 2018), had increased transcript levels by both treatments, which were significantly higher by O_3_ compared to NO_2_. Transcript levels for *RBOHF* (*RESPIRATORY BURST OXIDASE HOMOLOG F*), encoding a ROS producing enzyme, increased after O_3_ but decreased after NO_2_ treatment (**Figure 2**). This represented the most contrasting effect of the two gases.

Overall, four of five studied marker genes had increased transcript levels by both pollutants with O_3_ having a slightly stronger impact than NO_2_. There were no major differences between tolerant or sensitive accessions. For instance, the tolerant accession Ost-0 showed very similar transcript profiles as the sensitive accession Cvi-0. Taken together, **Figures 1**, **2** support the conclusion that O_3_ and NO_2_ trigger the expression of similar sets of defence- and cell death-related genes also in different *A. thaliana* accessions. However, the very contrasting regulation of *RBOHF* (increased transcript levels by O_3_ and decreased transcript levels by NO_2_), show that in addition to common signaling pathways induced by both gases, there is a mechanism by which *A. thaliana* can perceive and initiate signaling that is unique for O_3_ versus NO_2_.

### 3.3 O_3_- and NO_2_-induced leaf symptoms are similar between accessions

**Figure 3:**
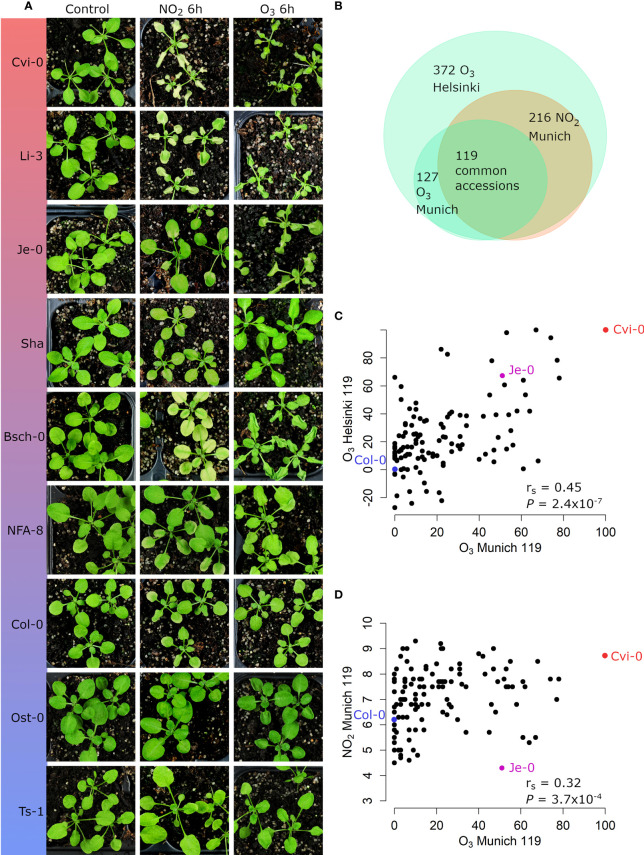
O_3_ injury and NO_2_ injury phenotypes of different accessions. **(A)** Images of clean air control, 6h of 10 ppm of NO_2_ treated and 6h of 350 ppb O_3_ treated accessions in Munich. **(B)** Overlap of accessions used in the different O_3_ and NO_2_ treatments. **(C)** O_3_ injury phenotypes (percentage of damaged leaves relative to Col-0) in Munich of the 119 common accessions are plotted against O_3_ injury phenotypes in Helsinki. **(D)** NO_2_ injury phenotypes (cumulative score) are plotted against O_3_ injury phenotypes in Munich. The O_3_ phenotypes are from two biological repeats of O_3_ fumigations (350 ppm, 6h), with 5 plants (Munich) or 8 plants (Helsinki) per accession (see **Supplementary Table S7** for the scores for each accession). O_3_ sensitivity was quantified as O_3_‐induced visible leaf injury (number of leaves with damage/total leaves, displayed as percentages and normalized to Col-0). The NO_2_ phenotypes are visible injury scores from three fumigations (10, 20 and 30 ppm) with 3 plants per accession. The cumulative score is on a scale from 3 to 15. Spearman’s correlation coefficients (r_s_) are presented on the bottom right corner **(C, D)**. The accessions Col-0 and Cvi-0, which were included in all treatments and repeats, are highlighted in blue and red, respectively.

Natural accessions show a wide range of O_3_ tolerance versus sensitivity (Brosche et al., 2010). As the transcriptome data (**Figure 1B**, **Supplementary Tables S5** and **S6**) has high overlap in O_3_ and NO_2_ induced biological mechanisms, we evaluated whether different accessions developed similar damage to O_3_ and NO_2_. Ts-1, Ost-0, and Col-0 were tolerant while Bsch-0, Li-3, and Cvi-0 were sensitive to both pollutants. NFA-8 and Shahdara had intermediate phenotypes, e.g. Shahdara was clearly sensitive to O_3_ in Helsinki conditions, less so in Munich and neither sensitive nor tolerant for NO_2_. Je-0 had a differential phenotype since it was rather tolerant to NO_2_ but clearly sensitive to O_3_ (**Figure 3A**).

We extended the investigation of O_3_- and NO_2_-related leaf phenotypes to large sets of accessions (**Supplementary Table S1**). Independent O_3_ damage screens took place in Helsinki where 372 accessions were treated for 6 h with 400 ppb O_3_ and in Munich where 127 accessions received 350 ppb O_3_ for 6 h. In both experiments results of the damage score (% of leaf area damaged) did not show normal distribution because tolerant accessions were overrepresented. In Munich, 216 accessions were treated with 10, 20, or 30 ppm NO_2_ for 1 h. This treatment scheme facilitated a fine-tuned and nearly normally distributed rating of NO_2_ leaf damage.

A subset of 119 accessions was common for all three experiments (**Figure 3B**), which allowed us to compare damage from O_3_ and NO_2_, as well as the comparison of O_3_ damage at two different facilities. Altogether, the tested accessions exhibited large phenotypic variation after fumigation with O_3_ or NO_2_ (**Figure 3**, **Supplementary Table S7**). Scatter plots revealed a significant correlation in the extent of lesion formation between both O_3_ experiments (Spearman’s correlation coefficient r_s_ = 0.45, *P* = 2.4 x 10^-7^, **Figure 3C**). Likewise, the phenotypes caused by the O_3_ and NO_2_ fumigations in Munich showed a good correlation (r_s_ =0.32, *P* = 3.7 x 10^-4^, **Figure 3D**) although several accessions had differential sensitivities to both pollutants including Je-0 (**Figure 3A**, see **Supplementary Table S7** for further examples).

### 3.4 Identification of candidate genes involved in phenotypic responses to O_3_ and NO_2_ using GWAS

**Figure 4:**
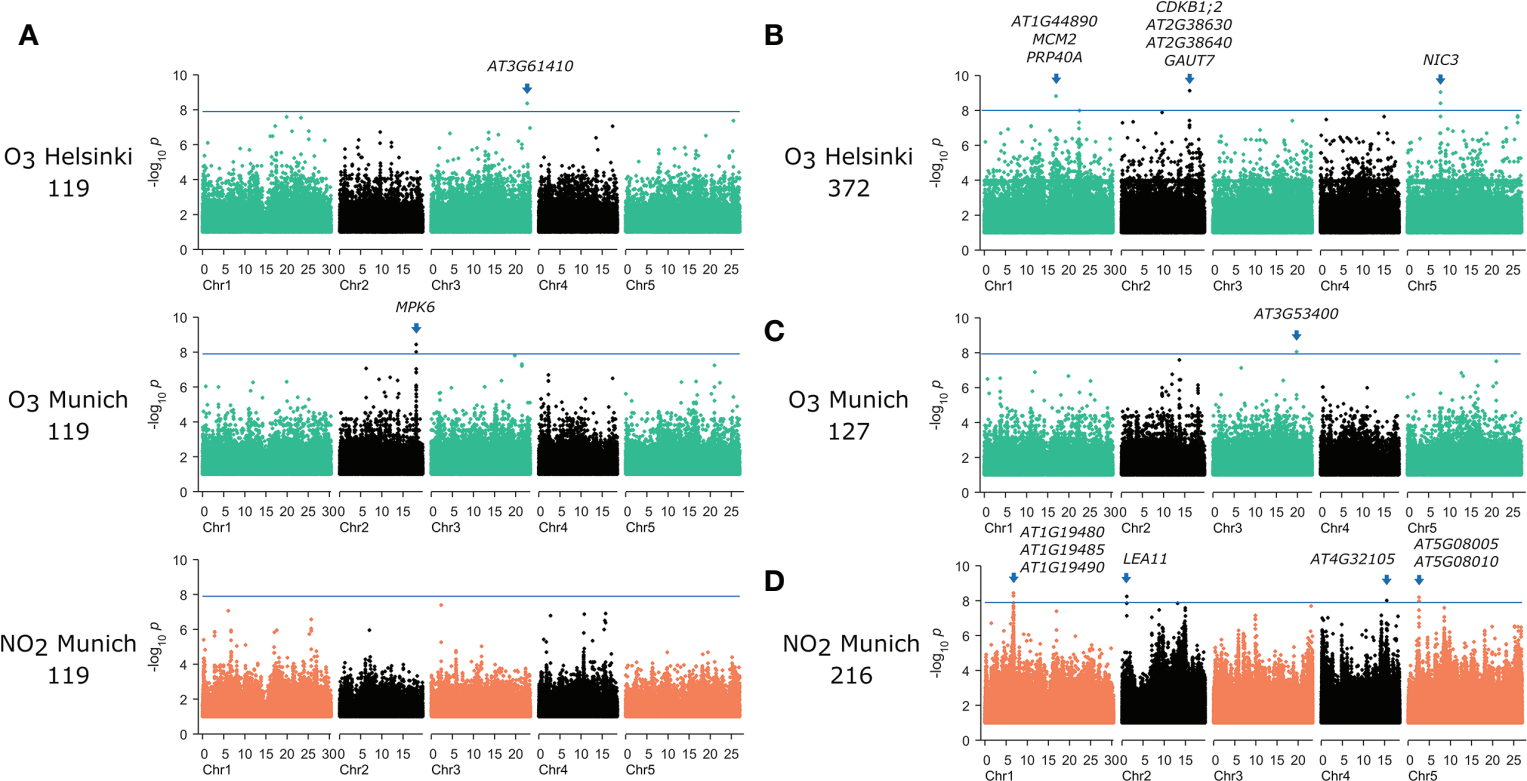
Manhattan plots of GWAS results. Results **(A)** from accelerated mixed model (AMM) analysis with the common set of 119 accessions in the three different experiments, **(B)** with 372 accessions treated with O_3_ in Helsinki, **(C)** 127 accessions treated with O_3_ in Munich and **(D)** results from non-parametric Kruskall-Wallis tests (KW) of 216 accessions treated with NO_2_. The horizontal line corresponds to Bonferroni corrected *P* < 0.05 significance threshold. Genes with significant SNPs are marked with an arrow. The SNPs are plotted according to their physical order in the chromosomes. The Mb distance is marked in below each chromosome.

We used the leaf damage scores for the 119 accessions common to all three fumigation screens to perform GWAS, and to identify and compare SNPs associated with O_3_- and NO_2_-induced phenotypes. From GWAS, the *P*-values are a measure of association strength between SNPs and leaf damage of the different accessions. The analysis included both the non-parametric Kruskal-Wallis (KW) test and the accelerated mixed model (AMM) analysis that corrects for population structure. As a final outcome, this led to the identification of genes containing SNPs - or being within high linkage disequilibrium (LD) of SNPs - that were significantly associated with the damage score (**Figure 4** and **Table 1**). Overall, Bonferroni-adjusted AMM statistics identified three significant SNPs (with -log_10_ *P*-values higher than 8) from the three O_3_ and NO_2_ experiments with the common 119 accessions (**Figure 4A**). In the Helsinki dataset, a single associated SNP was found in the 5’ UTR of *AT3G61410* coding for a U-box kinase family protein. Two of these SNPs were significantly associated with O_3_ induced leaf damage in the Munich dataset (**Figure 4A**), where the smallest *P*-value SNPs were in an intergenic region closest to a gene with unknown function *AT2G43795* and adjacent to *MPK6* (*MAP KINASE 6*).

Subsequently, GWAS was performed on each individual dataset, with full number of accessions phenotyped, which identified additional SNPs significantly associated with O_3_-induced leaf phenotypes (**Table 1**). The AMM analysis of the O_3_/Helsinki full dataset (372 accessions) uncovered several non-synonymous SNPs in the coding sequence of *NIC3* (*NICOTINAMIDASE 3*), two significant SNPs upstream of the 5’ UTR of *PRP40A* (*PRE-MRNA-PROCESSING PROTEIN 40A*), and one significant SNP upstream of *AT1G44890*. Localized between the latter two genes, *MCM2* (*MINICHROMOSOME MAINTENANCE 2*) exhibited a high linkage disequilibrium (LD) with the respective SNPs. Further SNPs were found in an intergenic region of chromosome 2 between the two genes *AT2G38630* and *AT2G38640*. All these SNPs had low minor allele frequencies (MAF) < 0.03, and thus represent rare SNPs in the studied set of accessions. AMM analysis of the O_3_/Munich full dataset (127 accessions) detected an additional SNP associated with the extent of O_3_-induced leaf damage in the 3’ UTR of *AT3G53400*. However, GWAS with the KW test did not result in the identification of SNPs in either O_3_ datasets (**Supplementary Table S8**).

GWAS of the full NO_2_ dataset with 216 accessions did not reveal any significant SNPs using AMM analysis (**Supplementary Table S8**). However, when applying the KW test tens of SNPs in chromosome 1 showed significant association with the leaf damage score. The smallest *P*-value SNPs were localized in genes coding for a DNA glycosylase (*AT1G19480*), Transducin/WD40 repeat-like superfamily (*AT1G19485*), and a basic-leucine zipper transcription factor (*AT1G19490*). Other significantly phenotype-associated SNPs were in the coding sequences of *AT2G03740* and *AT4G32105* and on chromosome 5 between the genes *AT5G08005* and *AT5G08010*.

We performed an additional GWAS with genotype data from a 250K SNP array (Kim et al., 2007; Atwell et al., 2010). Here, several genes, including *ABH1* (*ABA HYPERSENSITIVE 1*/*CAP-BINDING PROTEIN 80*) and *CNGC1* (*CYCLIC NUCLEOTIDE GATED CHANNEL 1*) were associated with O_3_ damage (**Supplementary Figure S2**, **Table 1**).

**Figure 5:**
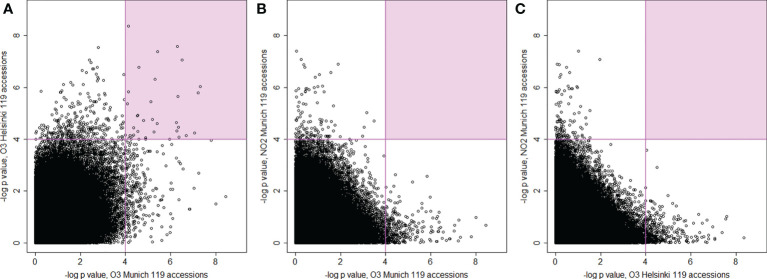
Scatterplots for the comparison and correlations between –log_10_ *P*-values of SNPs from genome-wide association analysis (AMM) between the three experiments with 119 common accessions. **(A)** plotted *P*-values from O_3_ fumigations conducted in Munich (x-axis) and in Helsinki (y-axis), **(B)** plotted *P*-values from O_3_ fumigations in Munich (x-axis) and NO_2_ fumigations (y-axis), **(C)** plotted *P*-values from O_3_ fumigations in Helsinki (x-axis) and NO_2_ fumigations (y-axis). The areas with the smallest *P*-values (–log_10_ *P*-value > 4) have coloured background.

In GWAS multiple testing correction is commonly used to provide fewer false positives (here Bonferroni adjustment). To allow comparisons between treatments, in subsequent analysis we also included SNPs with lower P-values. SNPs from the two O_3_ experiments revealed no common SNPs with -log_10_ *P*-values > 8, whereas 3 SNPs and 341 SNPs were shared with -log_10_ *P*-values > 6 and > 4 (**Figure 5A**, **Supplementary Table S8**). Altogether 47 (O_3_ Helsinki) and 63 (O_3_ Munich) SNPs showed -log_10_ *P*-values > 6 and 1716 and 1681 SNPs showed -log_10_ *P*-values > 4, respectively. Surprisingly, plotting the GWAS results of the NO_2_ experiment against those of the O_3_ experiments showed no shared SNPs with -log_10_ *P*-values > 4 (**Figures 5B, C**). In sum, the GWAS indicated very few genomic regions containing SNPs that have strong associations with O_3_ or NO_2_ leaf damage. Instead, there were many weak associations with O_3_ and NO_2_ sensitivity, and no overlap of SNPs between O_3_ and NO_2_ treatments.

### 3.5 Phenotyping of T-DNA insertion lines and O_3_ sensitive mutants

**Figure 6:**
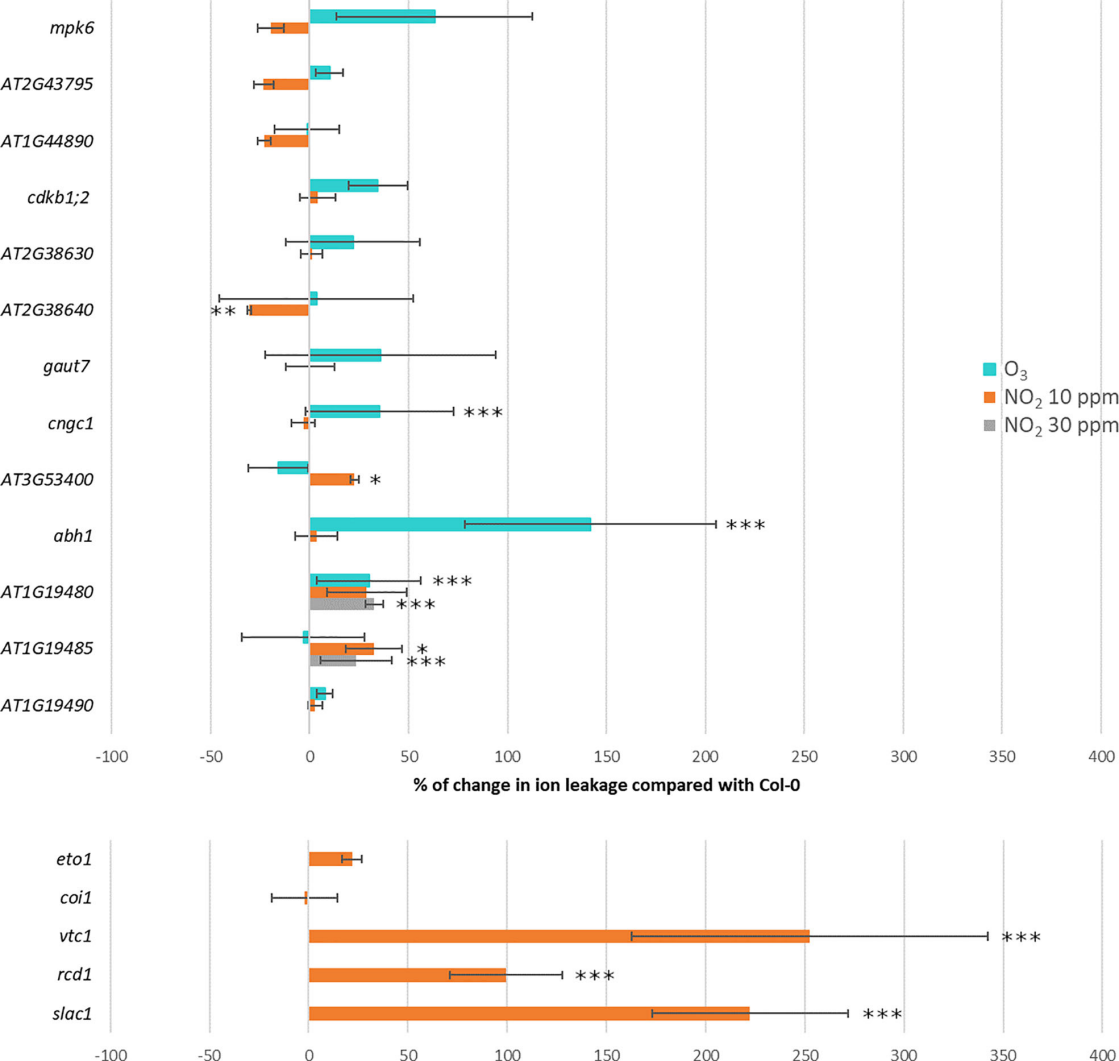
Percentage of change in ion leakage in GWAS candidate mutants (the upper part) and known O_3_ sensitive mutants (the lower part of the figure) relative to Col-0. Treatment with 350 ppb of O_3_ for 6h is marked in turquoise, 10 ppm of NO_2_ for 6h in orange and 30 ppm of NO_2_ for 1h in gray. Statistical significance is marked with asterisks: ***P < 0.001, **P < 0.01 and *P < 0.05.

We identified T-DNA insertion mutants for several GWAS candidate genes (for location of T-DNA inserts within the genes, see **Supplementary Figure S3**). We mainly selected based on *P*-values (**Table 1**), but for *P*-values with lower significance we also included candidate genes with biological functions related to plant defense responses. We measured the extent of damage as relative ion leakage after treatment for 6 h with 350 ppb O_3_ or 10 ppm NO_2_ (**Table 1**, **Figure 6** and original data in **Supplementary Table S9**). Relative ion leakage provides a better presentation of the data as the O_3_ experiment was done in Helsinki and the NO_2_ experiment in Munich. Col-0 was set as the baseline as all the mutants were in Col-0 background. Therefore, mutant line more sensitive than Col-0 have positive ion leakage values and more tolerant negative values.

Of the selected O_3_ candidate genes, *abh1* and *cngc1* were O_3_ sensitive (**Supplementary Figure S4**), whereas *At3g53400* was NO_2_ sensitive and *At2g38640* more tolerant to NO_2_ than the background accession Col-0. For *mpk6* and five other mutants no significant change in ion leakage was observed compared to Col-0. Among the three NO_2_ candidate genes tested, mutant lines with T-DNA insertions in *AT1G19480* and *AT1G19485* were O_3_ and NO_2_ sensitive, respectively. Next, we asked whether higher NO_2_ concentrations would improve the detection of altered mutant phenotypes. Therefore, we exposed Col-0 to 30 ppm NO_2_ which resulted in 50-65% ion leakage. With this treatment two tested NO_2_ candidate were shown to be NO_2_ sensitive (*AT1G19480* and *AT1G19485*). While we could measure statistically significant changes in ion leakages for some of the mutants selected from GWAS candidates, overall these changes were small compared to the cell death found in O_3_ or NO_2_ sensitive mutants in Col-0 background (Brosche et al., 2014; Xu et al., 2015a; Kasten et al., 2016). This is in line with the overall GWAS results, i.e. O_3_ and NO_2_ damage in natural accessions are associated with many small effect loci rather than major effect loci.

Previously published O_3_ sensitive mutants include ascorbate-deficient *vtc1* (Conklin et al., 1996), ethylene overproducer *eto1* (Rao et al., 2002), transcriptional co-regulator *rcd1* (Overmyer et al., 2000; Brosche et al., 2014), JA receptor *coi1* (Xu et al., 2015a) and guard cell S-anion channel *slac1* (Vahisalu et al., 2008). To allow a direct comparison of damage in mutants for GWAS candidates with the previously identified mutants, we used five O_3_ sensitive mutants and performed ion leakage with 10 ppm NO_2_ (we did not repeat the O_3_ damage for these mutants, as they are extensively characterized in previous publications). The *rcd1*, *vtc1* and *slac1* mutants were strongly NO_2_ sensitive, whereas *eto1* and *coi1* were not (**Figure 6**). Kasten et al. (2016) tested 30 ppm NO_2_ dose for these mutants and *eto1* showed a NO_2_ sensitivity phenotype whereas *coi1* was similar to Col-0. In sum, the phenotyping of GWAS T-DNA mutant lines and O_3_ sensitive mutants indicated that O_3_- and NO_2_-induced leaf damage was controlled by partially overlapping sets of genes. However, there were also exceptions, as the O_3_ sensitive *coi1* was tolerant to NO_2_.

## 4 Discussion

O_3_ has emerged as a large threat to agricultural production in Asia, including wheat and rice (Feng et al., 2022b). Further understanding of the genetic basis of plant sensitivity to air pollutants is required to guide breeding programs aimed at providing plants with improved tolerance (Frei, 2015). O_3_ tolerance and sensitivity traits are present in different genotypes and mapping populations of wheat (Feng et al., 2022a), maize (Choquette et al., 2019), rice (Frei et al., 2008) as well as *A. thaliana* [this study (Brosche et al., 2010; Jakobson et al., 2016; Morales et al., 2021)]. Combined genetic analysis with physiological traits has identified some of the mechanisms behind differential O_3_ sensitivity; for example, a mutation that leads to more open stomata in the *A. thaliana* accession Cvi-0 leads to higher O_3_ uptake and O_3_ damage (Brosche et al., 2010; Jakobson et al., 2016). Variation in photosynthetic parameters offers another possibility to follow O_3_ sensitivity traits (Choquette et al., 2019; Morales et al., 2021). Here we used the two air pollutants O_3_ and NO_2_ in *A. thaliana* to further understand their impact on regulation of signaling pathways and PCD. Ultimately, this could provide new mechanisms for tolerance that could be targeted in plant breeding programs.

### 4.1 O_3_- versus NO_2_-induced transcriptional responses

Exposure of Col-0 to 10 ppm NO_2_ for 1 h or 350 ppb O_3_ for 2 h resulted in massive transcriptional changes (Xu et al., 2015b; Mayer et al., 2018). Both gases regulated largely overlapping sets of genes involved in ROS, ethylene signaling, pathogen resistance, and cell death (**Figure 1B**, **Supplementary Tables S5** and **S6**). This is consistent with the rapid accumulation of ROS, NO, and ethylene in O_3_-exposed tobacco (Ederli et al., 2006). ROS and RNS bursts were also observed after NO_2_ fumigation of *A. thaliana* (Kasten et al., 2016). Plants produce ROS and RNS molecules as signaling molecules to regulate local and systemic long distance defence responses (Wang et al., 2013; Waszczak et al., 2018; Hancock and Neill, 2019; He et al., 2022). Accordingly, O_3_ and NO_2_ both increase transcript levels for pathogen responsive genes (Xu et al., 2015a; Mayer et al., 2018). In sum, O_3_ and NO_2_ probably act both as donors (i.e. to generate) as well as inducers of simultaneous ROS and RNS bursts that ultimately lead to the onset of defence responses.

We focused on the GO category cell death and performed Bayesian hierarchical clustering with O_3_ and various NO related treatments (**Figure 1A**). This revealed similar expression profiles in both O_3_ tolerant (Col-0, C24) and sensitive accessions (Cvi-0, Te). To corroborate this finding, we analysed transcript levels for five cell death related marker genes in a side-by-side comparison of nine accessions treated with 350 ppb O_3_ or 10 ppm NO_2_ for 2 h (**Figure 2**). Both gases increased transcript levels of *FMO1*, *GRX480*, *CEJ1*, and *RAP2.6* that function in defence signaling (Krishnaswamy et al., 2011; Caarls et al., 2015; Hartmann et al., 2018). Importantly, *RBOHF* was differentially regulated with increased transcript levels after O_3_, but decreased after NO_2_ treatment (**Figure 2**). RBOHF, but not RBOHD, was previously implicated as a regulator of O_3_ cell death (Xu et al., 2015a), hence differential use of ROS produced from RBOHs could be a mechanism to regulate cell death in response to different signals. The transcript level variation between the accessions was independent of their O_3_ or NO_2_ sensitivity. For instance, the O_3_ tolerant accession Ts-1 showed comparable gene regulation as the sensitive accession Cvi-0. Taken together, **Figures 1**, **2** support the conclusion that O_3_ and NO_2_ regulate the expression of largely overlapping sets of defence-related genes also in different *A. thaliana* accessions. This implies that the mechanism(s) used by *A. thaliana* to perceive ROS (O_3_) and RNS (NO_2_) are conserved in genetically distant *A. thaliana* accessions. Initial NO perception in *A. thaliana* takes place *via* targeted degradation of group VII ethylene response factors (ERFs) (Gibbs et al., 2014). Further down-stream signaling is proposed to be mediated by several other transcription factors, including RAP2.6 (Imran et al., 2018; Leon et al., 2020). Since *RAP2.6* transcript levels were enhanced by both O_3_ and NO_2_, it represents a target for both ROS and RNS signaling.

There was one exception to the common transcriptional response by O_3_ and NO_2_, *RBOHF* was differentially regulated, with increased transcript levels after O_3_, but decreased after NO_2_ treatment (**Figure 2**). In both plant stress, PCD and developmental responses, the RBOH proteins produce superoxide as signaling molecules (Castro et al., 2021). Out of the ten *A. thaliana* RBOHs, RBOHD and RBOHF are the main producers of ROS during various aspects of defence signaling (Castro et al., 2021). RBOHF was previously implicated as a regulator of O_3_ cell death (Xu et al., 2015a), and in ROS transcriptional responses (Willems et al., 2016). ROS from RBOHD and RBOHF are also required to execute cell death in pathogen HR and progression of cell death lesions (Torres et al., 2002; Torres et al., 2005). Differential use of ROS produced from distinct RBOHs could be a mechanism to regulate cell death in response to different signals. The opposite regulation *RBOHF* transcript levels were present in all tested accessions (**Figure 2**), this means that *A. thaliana* can activate distinct signaling pathways from O_3_ versus NO_2_. Further analysis of the promoter region of *RBOHF* could lead to identification of e.g. transcription factor(s) and promoter elements that regulate this specific signaling pathway with contrasting regulation by O_3_ and NO_2_.

### 4.2 Use of GWAS to identify genes regulating O_3_ and NO_2_ cell death

Improvement of plant varieties by breeding could help to reduce yield losses due to pollutant-induced leaf damage. Plant breeding can be guided by the results from quantitative trait loci (QTL) or association mapping studies (Frei, 2015; Ueda et al., 2015; Begum et al., 2020). Here, we used *A. thaliana* natural accessions to identify genes involved in regulation of lesion formation after O_3_ and NO_2_ exposure. We performed GWAS on two independent O_3_ screens and one NO_2_ screen. 119 accessions were investigated in all three experiments. Initial GWAS runs focused only on 119 accessions assuming that at least some genetic loci would be associated with both O_3_- as well as NO_2_-induced leaf phenotypes. However, this approach did not reveal any significant SNPs shared between the O_3_ and NO_2_ screens (**Figure 4**).

Even with standardized growth conditions, growth and molecular responses of *A. thaliana* show differences between different laboratories (Massonnet et al., 2010). To explore these differences, we performed the phenotyping of O_3_ tolerance at two different facilities (Helsinki and Munich). Replication of GWAS is common in human studies but relatively rare in other organisms. As we repeated our O_3_ GWAS at two different facilities, this can give some insight into what to expect from GWAS replications in *A. thaliana*. There was some phenotypic variation, but also a significant positive correlation between the same accessions in the two O_3_ experiments. Importantly, we found noticeable overlap in small *P*-value GWAS SNPs between the two O_3_ datasets (**Figure 5A**, 341 shared SNPs with -log_10_ *P*-values > 4), which emphasizes that *A. thaliana* has a robust genetic response to O_3_. For instance, the most significant SNP for the common 119 accessions in Helsinki (*AT3G61410*) had also a small *P-*value in Munich (–log_10_ *P*-value = 4.1). Furthermore, by combining results from shared small *P*-value SNPs from GWAS replications, additional candidate genes can be selected for further study. This would be especially useful as many traits of interest are controlled by several small effect genes, for which SNPs may not reach significance level after correction for multiple testing.

Subsequently, we analysed the datasets individually and we found 12 genomic loci significantly associated with the extent of leaf damage (**Figure 5** and **Table 1**) but again none of these loci was shared between the NO_2_ and O_3_ screens. GWAS for O_3_ tolerance has previously been performed in rice (Ueda et al., 2015), which identified 16 loci with rather weak phenotypic associations (*P*<0.0001). Similarly, in a study with 150 wheat varieties statistics indicated weak associations between SNPs and O_3_-induced phenotypes because the determined *P*-values were rarely below 0.0001 (Begum et al., 2020). Classical QTL mapping studies indicated that several genes in different chromosomal locations control O_3_ sensitivity in *A. thaliana* (Brosche et al., 2010; Xu et al., 2015b; Jakobson et al., 2016). These findings argue for O_3_ and NO_2_ phenotypes being determined by multiple small-effect loci that are detectable either by QTL-mapping families with contrasting parental phenotypes (which gives the possibility to identify rare alleles in *A. thaliana* populations) or by using large numbers of accessions in GWAS to improve statistical sensitivity (and more likely to identify common alleles). Accordingly, GWAS with O_3_ screening data from 127 accessions (Munich screen) resulted in the identification of only a single significantly associated genomic region (only SNPs in one gene in high linkage disequilibrium with the most significant SNP) whereas data from 372 accessions (Helsinki screen) revealed 3 associated genomic regions (with SNPs in 8 genes in high LD with the most significant SNPs). Hence, future screens should include as many of the >1000 sequenced accessions (Alonso-Blanco et al., 2016), as possible to identify more genes linked to O_3_- and NO_2_-associated phenotypes and assess whether there is reasonable overlap in the genetics underlying responses to both pollutants. However, with the current datasets we could not find significantly, or even among small *P*-value (**Figures 5B, C**), overlapping SNPs and thus it appears that O_3_ versus NO_2_ induced lesions are controlled by different genetic loci.

Based on the GWAS results we screened 13 T-DNA knock-out mutants for candidate genes (**Table 1** and **Figure 6**). Five mutants were sensitive and one mutant tolerant as compared to wild-type plants. *ABH1* is a gene coding for a nuclear mRNA cap-binding protein that participates in abscisic acid signaling and mRNA processing (Hugouvieux et al., 2001; Laubinger et al., 2008). CNGC1 is an ion channel likely functioning in calcium signaling (Sunkar et al., 2000). The mutant for *At1g19480* was sensitive to both 10 ppb O_3_ as well as 30 ppm NO_2_ (**Figure 6**). AT1G19480 might be involved in DNA repair that is an important process in ROS-exposed plants (Nisa et al., 2019). *CPuORF46* (*AT3G53400*) encode an upstream open reading frame (uORF) which can conditionally regulate translation of the main ORF, and has been shown to be responsive to heat stress (Causier et al., 2022). Future research can characterize the physiological mechanisms causing the NO_2_- and O_3_-phenotypes in these mutants including measurements of the stomatal aperture, transcriptional changes, stress hormone levels, and antioxidant status.

Both O_3_ and NO_2_ cause formation of PCD lesions, with similarities to pathogen HR (Overmyer et al., 2005; Kasten et al., 2016). To further compare how O_3_ and NO_2_ regulate lesion formation we used the O_3_ sensitive *vtc1* and *slac1* (Conklin et al., 1996; Vahisalu et al., 2008). Both mutants displayed stronger cell death and increased ion leakage upon exposure to NO_2_ than any other mutant tested in the current study (**Figure 6B**). The *vtc1* mutant is ascorbate-deficient whereas *slac1* has an increased stomatal aperture and impaired responses to signals leading to stomatal closure. Hence, antioxidant levels and stomatal regulation restricting entry of air pollutants into the plant are important common determinants of both O_3_ and NO_2_ toxicity.

**Figure 7:**
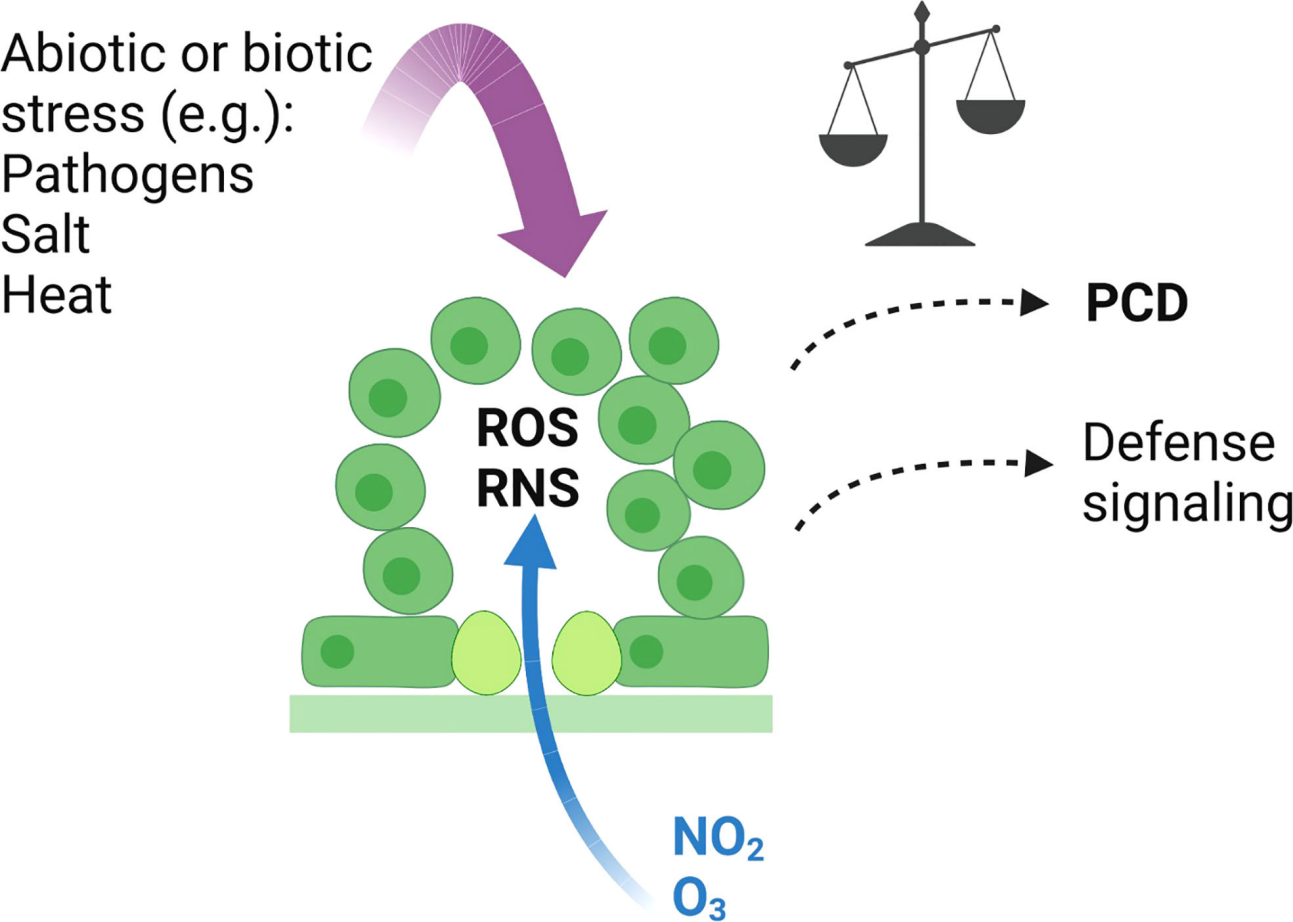
Signalling events initiated by O_3_ and NO_2_. O_3_ and NO_2_ enter the plant through stomata, and in the apoplast break down into ROS (including H_2_O_2_ and O_2_^.-^), and RNS (including NO). This initiates various signalling pathways leading to large-scale transcriptional changes, where O_3_ and NO_2_ mostly regulate the same set of genes (**Figure 1**). In sensitive genotypes, PCD is activated leading to leaf damage. Whether PCD is activated depends on the balance of ROS and RNS signalling (Delledonne et al., 2001; Ahlfors et al., 2009), the antioxidant capacity to remove ROS and RNS (Conklin et al., 1996; Kasten et al., 2016), hormone signalling (Xu et al., 2015a; Kasten et al., 2016) and other to be discovered mechanisms. Placement of new candidate PCD regulators identified from GWAS into signalling pathways will require additional studies. The application of O_3_ and NO_2_ leads to a clearly defined subcellular source of ROS and RNS in the apoplast. Multiple other abiotic and biotic stresses also lead to active ROS and RNS signalling (Wendehenne et al., 2014; Waszczak et al., 2018; He et al., 2022; Hussain et al., 2022). However, in response to stress the production site of ROS and RNS can come from multiple parts of the cell including apoplast, chloroplast and mitochondria (Waszczak et al., 2018; Hancock and Neill, 2019; Castro et al., 2021). Created with BioRender.com.

Overall, using O_3_ and NO_2_ we show that ROS and RNS have largely overlapping transcriptional responses, but at the same time, they also have distinct signaling roles as exemplified by the contrasting transcriptional regulation of *RBOHF*. Similarly, mutant analysis also revealed mutants that were sensitive to both gasses as well as mutants sensitive to only one gas. No common SNPs were identified by the GWAS analysis suggesting that natural variation in the strength of PCD induced by the gasses is caused by different small effect genes. Due to the relative simplicity of applying O_3_ or NO_2_ to plants in controlled growth conditions, further studies with these gasses will allow dissection of ROS and RNS signaling pathways in plant stress and PCD regulation. Identification of novel PCD regulators can aid in development of strategies to combat the negative effects of air pollution. **Figure 7** illustrates connections between O_3_ and NO_2_ and other stresses that can initiate defence signaling and PCD.

## Data availability statement

The original contributions presented in the study are included in the article and supplementary material. Raw data used for re-analysis of microarray and RNA-seq data are described in detail in Materials and Methods and in **Supplementary Table S2**.

## Author contributions

JL, FG, JD, and MB initiated and designed the experiments. JL and FG performed the experiments. JL, FG, EX, LM, and MB analyzed the data. JL, FG, and MB wrote the manuscript and all authors commented and approved the submitted version.

## Funding

For funding, in Helsinki, we acknowledge a grant number 307335 from the Academy of Finland, for Centre of Excellence in Molecular Biology of Primary Producers. The gas exposure screens in Munich were supported by the European Plant Phenotyping Network (EPPN, Project No. 28443).

## Acknowledgments

We would like to acknowledge Tuomas Puukko for excellent qPCR lab work and Omid Safronov for help with analysis of RNA-seq data. Minna Koskela, Valtteri Norkola, Anna Huusari, Valtteri Lehtonen and Adrien Gauthier are thanked for assistance with plants. Hans Lang and Andreas Albert are thanked for technical help with the experiments. Lauri Vaahtera and Folmer Bokma are acknowledged for help with the figures.

## Conflict of interest

The authors declare that the research was conducted in the absence of any commercial or financial relationships that could be construed as a potential conflict of interest.

## Publisher’s note

All claims expressed in this article are solely those of the authors and do not necessarily represent those of their affiliated organizations, or those of the publisher, the editors and the reviewers. Any product that may be evaluated in this article, or claim that may be made by its manufacturer, is not guaranteed or endorsed by the publisher.
