# Supplementary material for: Ozone and nitrogen dioxide regulate similar gene expression responses in Arabidopsis but natural variation in the extent of cell death is likely controlled by different genetic loci
Source: Front Plant Sci. 2022 Oct 19;13:994779. doi: 10.3389/fpls.2022.994779 (PMC9627343; doi:10.3389/fpls.2022.994779)
Supplement: Supplementary file 10 [file DataSheet_1.pdf]

# *Supplementary Material*

## 1 Supplementary Methods – Analysis of RNA-seq data

RNA-seq data for Col-0 exposed to $O_3$ (Xu *et al.*, 2015) and to the NO-donor S-nitrosocysteine (Hussain *et al.*, 2016) were re-analyzed in R. Removal of adapter sequences and trimming and cropping of the reads were done using Trimmomatic-0.33 (Bolger *et al.*, 2014) in single-end mode. The bases with a Phred quality score < 20 were trimmed from the ends of the reads, and the reads shorter than 30 bases were removed from the analysis (-phred33, TRAILING:20 and MINLEN:30). Filtered reads were mapped to the *A. thaliana* reference AtRTD2 (Zhang *et al.*, 2017) using Kallisto V-0.43.0 (CMD:quant) (Bray *et al.*, 2016) with 4000 bootstrap sets. The final count table for each biological replicate was obtained as the mean of the bootstrap runs. Counts were normalized using the default Trimmed Mean of M-values and genes with less than five normalized counts were removed. The normalized counts table was analyzed for differential gene expression with edgeR 3.24.3 (Robinson *et al.*, 2010). The glmLRT (McCarthy *et al.*, 2012) method was used to fit the statistical model separately to data from each genotype in edgeR. Selected pairwise contrasts were assessed with method decideTestsDGE using Benjamini-Hochberg false discovery rate correction of *P*-values, with FDR ≤ 0.05 logFC > $\log_2(1.5)$ or logFC < $-\log_2(1.5)$ as thresholds.

## 2 Supplementary Figures

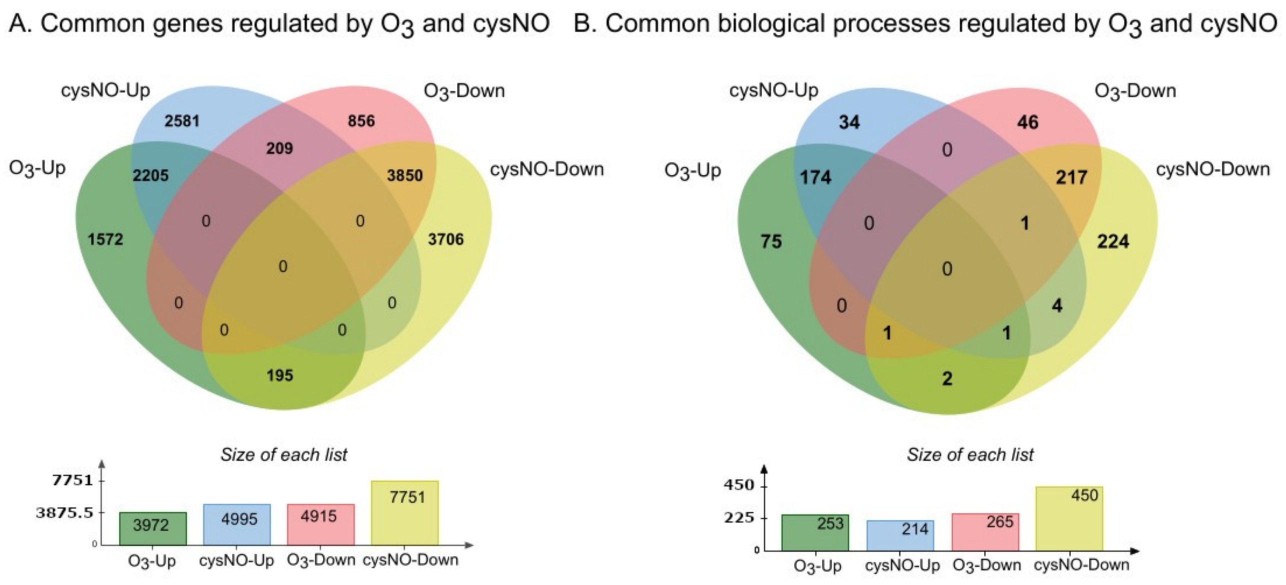


**Supplementary Figure 1.** Comparison of differentially expressed genes (A) and enriched GO categories in RNAseq experiments after $O_3$ treatment and treatment with S-nitrosocysteine (cysNO).

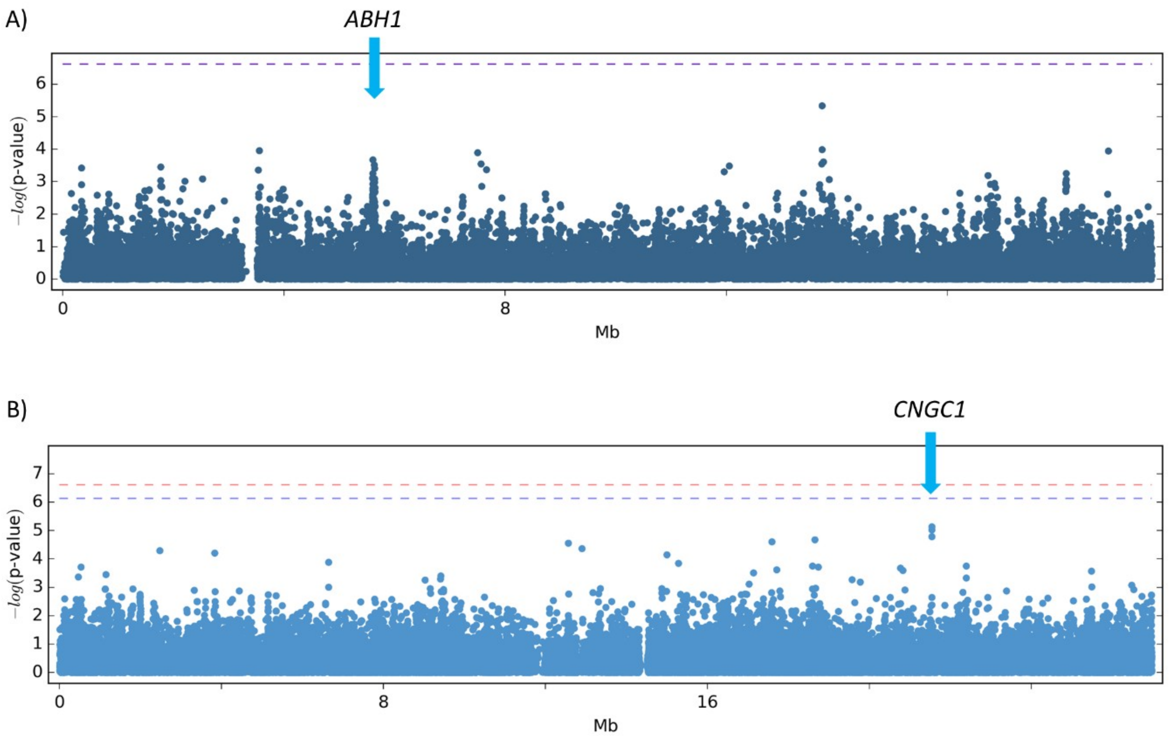


**Supplementary Figure 2.** Manhattan plots of GWAS results with 250K SNP chip for $O_3$ data (A) from Munich, 127 accessions, KW analysis, chromosome 2, (B) from Helsinki, 372 accessions, AMM analysis, chromosome 5. The horizontal lines represent 5% significance thresholds determined by Benjamini-Hochberg multiple testing procedure (A, and blue dashed line in B) or by Bonferroni correction (red dashed line in B).

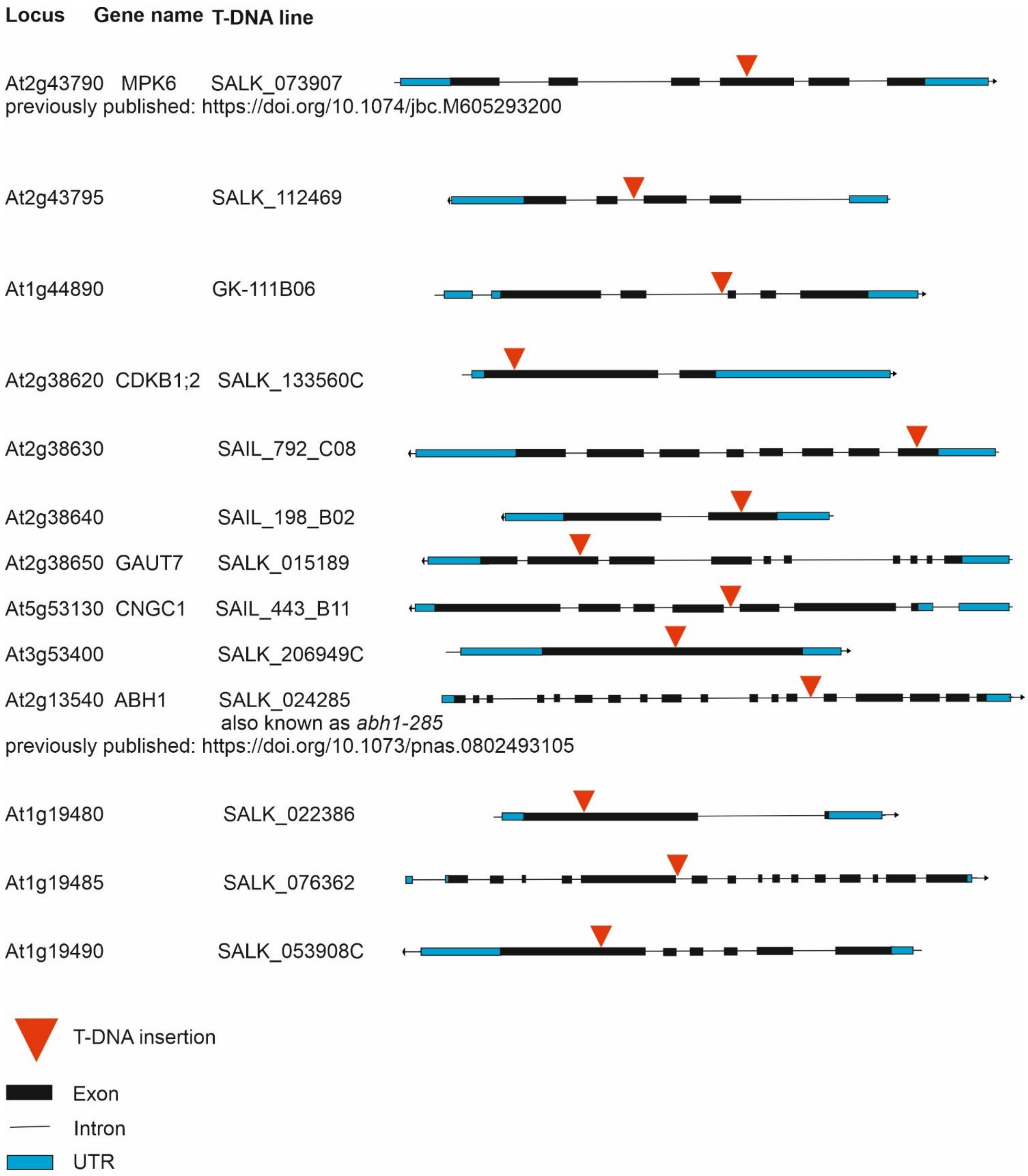


**Supplementary Figure 3.** Schematic representation of the T-DNA lines used for quantification of cell death after $O_3$ and $NO_2$ treatments. The gene models are not in scale to illustrate the actual sizes of the coding regions, the purpose is to illustrate the locations of T-DNA insertions (red triangles).

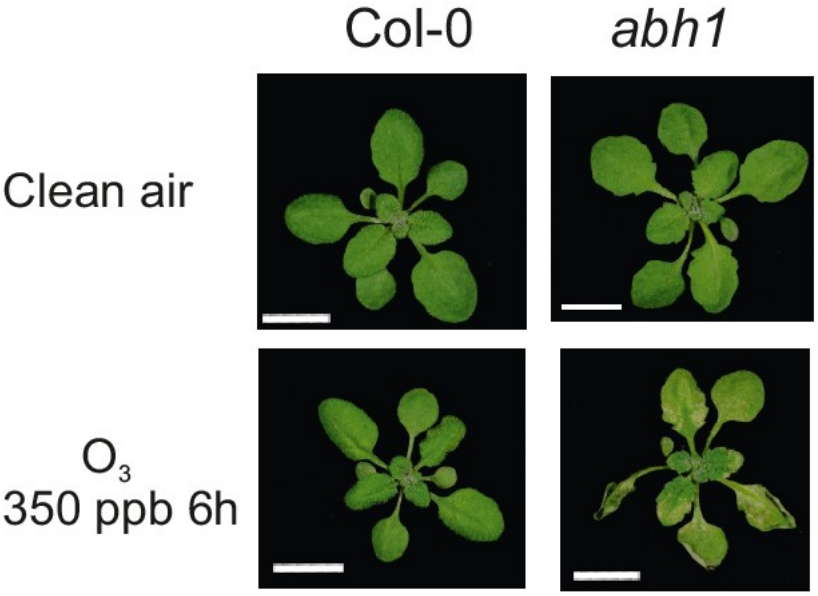


**Supplementary Figure 4**. Representative pictures of 3.5 weeks old Col-0 and *abh1* plants exposed to clean air and ozone ($O_3$): 350 nmol mol$^{-1}$ ozone during 6h. Pictures were taken 24h after the $O_3$ exposure was finished (scale bar 1cm).

## 3 List of supplementary tables

**Supplementary Table S1.** Natural accessions of *Arabidopsis thaliana* used in the $O_3$ and $NO_2$ experiments.

**Supplementary Table S2.** The microarray and RNAseq datasets involved in Bayesian hierarchical clustering of gene expression array data for $O_3$ and NO/$NO_2$ related treatments or mutants.

**Supplementary Table S3.** qPCR primer sequences and amplification efficiencies.

**Supplementary Table S4.** T-DNA mutant lines used for the ion leakage experiments. Name of the locus, mutation type and primers used for identifying homozygous mutants are listed.

**Supplementary Table S5.** Differentially expressed genes of $O_3$ RNAseq and $NO_2$ microarray data.

**Supplementary Table S6.** Differentially expressed genes and enriched GO categories in $O_3$ and CysNO RNA-seq experiments.

**Supplementary Table S7.** $O_3$ and $NO_2$ damage phenotypes in *A. thaliana* accessions from $O_3$ exposures in Munich and Helsinki, and $NO_2$ exposures in Munich.

**Supplementary Table S8.** A list of small P-value SNPs from $O_3$ and $NO_2$ GWAS with parametric (AMM) and non-parametric (KW) model.

**Supplementary Table S9.** Raw data from ion leakage measurements with T-DNA mutants treated with $O_3$ or $NO_2$.

## References

Bolger, AM., Lohse, M., Usadel, B. (2014) Trimmomatic: a flexible trimmer for Illumina sequence data. Bioinformatics 30, 2114–2120. https://doi.org/10.1093/bioinformatics/btu170

Bray, NL., Pimentel, H., Melsted, P., Pachter, L. (2016) Near-optimal probabilistic RNA-seq quantification. Nature Biotechnology 34, 525–527. doi.org/10.1038/nbt.3519.

Hussain, A., Mun, B.-G., Imran, Q.M., Lee, S.-U., Adamu, T.A., Shahid, M., et al. (2016). Nitric Oxide Mediated Transcriptome Profiling Reveals Activation of Multiple Regulatory Pathways in Arabidopsis thaliana. *Frontiers in Plant Science* 7**,** 975. doi: 10.3389/fpls.2016.00975.

McCarthy, D. J., Chen Y., Smyth, G. K. (2012). Differential expression analysis of multifactor RNA-Seq experiments with respect to biological variation. Nucleic Acids Research, 40, 4288–4297. https://doi.org/10.1093/nar/gks042

Robinson, M.D., McCarthy, D.J., Smyth, G.K. (2009) edgeR: a Bioconductor package for differential expression analysis of digital gene expression data. Bioinformatics 26, 139–140. https://doi.org/10.1093/bioinformatics/btp616

Xu, E., Vaahtera, L., Horak, H., Hincha, D.K., Heyer, A.G., and Brosche, M. (2015b). Quantitative trait loci mapping and transcriptome analysis reveal candidate genes regulating the response to ozone in Arabidopsis thaliana. *Plant Cell and Environment* 38(7)**,** 1418-1433. doi: 10.1111/pce.12499.

Zhang, R., Calixto, C.P.G., Marquez, Y., Venhuizen, P., Tzioutziou, N.A., Guo, W., Spensley, M., Entizne, J.C., Lewandowska, D., Ten Have, S., et al.  (2017) A high quality Arabidopsis transcriptome for accurate transcript-level analysis of alternative splicing. Nucleic Acids Research 45, 5061–5073. https://doi.org/10.1093/nar/gkx267
